# Preoperative Noble and Underwood Score in Colorectal Cancer Surgery: A Stable Prognostic Marker Even among Tumor Marker–Negative Cases

**DOI:** 10.31662/jmaj.2026-0107

**Published:** 2026-07-03

**Authors:** Masayuki Urabe, Masaya Hiyoshi, Mami Suzuki, Takahiro Fukai, Yui Hasegawa, Emi Terai, Yoshitaka Kiya, Goki Morizono, Toshiyuki Watanabe, Yojiro Hashiguchi

**Affiliations:** 1 Gastrointestinal Surgery Division, Department of Surgery, Japanese Red Cross Omori Hospital, Tokyo, Japan

**Keywords:** colorectal cancer, Noble and Underwood score, NUn score, prognosis, surgery

## Abstract

**Introduction:**

Reliable and widely applicable tools for survival risk stratification in colorectal cancer (CRC) remain to be established. Although the Noble and Underwood (NUn) score, comprising white blood cell count, C-reactive protein, and serum albumin, has been reported to possess prognostic relevance in some malignancies, its role in CRC has yet to be elucidated. We retrospectively evaluated the association between the preoperative NUn score and survival outcomes in patients with resectable CRC.

**Methods:**

A total of 608 consecutive patients who underwent curative resection for CRC were analyzed. The impact of the preoperative NUn score on overall survival (OS) and relapse-free survival (RFS) was assessed using Cox proportional hazards models. Optimal cutoff values were determined using X-tile software.

**Results:**

The median follow-up period was 65 months. Kaplan-Meier analyses demonstrated that the preoperative NUn score effectively stratified OS and RFS, including among tumor marker–negative cases. In univariate analyses, a high NUn score was significantly associated with worse OS and RFS. Multivariate analyses adjusted for clinically relevant factors, including tumor marker positivity, confirmed that a high NUn score independently predicted worsened OS (hazard ratio [HR] 2.06, 95% confidence interval [CI] 1.24-3.41, p = 0.016) and RFS (HR 1.66, 95% CI 1.09-2.53, p = 0.011). These independent associations remained robust when the NUn score was analyzed as a continuous variable.

**Conclusions:**

The preoperative NUn score independently predicted survival outcomes in resectable CRC and may serve as a practical tool to support individualized therapeutic decision-making.

## Introduction

Colorectal cancer (CRC) is the third most commonly diagnosed malignancy worldwide, accounting for 9.6% of all cancers and causing more than 900,000 deaths annually ^(1)^. With ongoing population aging and lifestyle changes, including rising obesity rates and dietary shifts, its incidence is expected to increase further, making CRC a major public health concern ^(2)^. Curative treatment is primarily based on surgical resection, often combined with multimodal strategies such as chemotherapy, radiotherapy, and immunotherapy; however, outcomes remain suboptimal ^(3)^. Therefore, effective preoperative risk stratification remains imperative. The ability to predict adverse prognosis would enable risk-adapted surgical strategies and more appropriate selection of additional therapies.

The prognosis of CRC is highly heterogeneous and cannot be fully explained by tumor-related factors alone, such as those represented by the tumor-node-metastasis (TNM) classification. Although tumor markers such as carcinoembryonic antigen (CEA) and carbohydrate antigen 19-9 (CA19-9) provide clinically valuable information, they do not fully capture the biological aggressiveness of the tumor. In contrast, accumulating evidence suggests that the systemic inflammatory response plays a central role in carcinogenesis and tumor progression. Host inflammatory and immune-nutritional status is closely associated with patient survival ^(4)^, and accordingly, numerous surrogate markers have been proposed to quantify these systemic conditions ^(5)^. However, many require complex calculations incorporating multiple perioperative variables, which limits their practicality and routine clinical applicability.

The Noble and Underwood (NUn) score, originally reported as a predictor of postoperative complications in esophageal cancer ^(6)^, is a simple inflammation- and nutrition-related index derived from white blood cell (WBC) count, serum albumin, and C-reactive protein (CRP). More recently, the NUn score has been shown to have prognostic value in gastric and lung cancers, outperforming several established markers ^(7), (8)^. However, its prognostic significance in CRC has not yet been evaluated. Based on the hypothesis that the NUn score may serve as a robust prognostic indicator in CRC, we retrospectively investigated the association between the preoperative NUn score and survival outcomes in patients undergoing curative resection for CRC.

## Materials and Methods

### Study population

We retrospectively reviewed all patients who underwent curative surgical resection for histologically confirmed CRC at our institution between January 2009 and December 2022. A total of 608 consecutive patients with stage I-III CRC were included in the final analysis after applying the following exclusion criteria: presence of distant metastasis, nonadenocarcinoma histology, preoperative endoscopic resection, neoadjuvant chemotherapy/chemoradiotherapy, postoperative follow-up of less than 3 months, simultaneous resection of another malignancy, and insufficient preoperative data.

Because the analysis was based exclusively on anonymized clinical data obtained during routine medical care, the requirement for written informed consent was waived. The study protocol conformed to the Declaration of Helsinki and the Ethical Guidelines for Medical and Health Research Involving Human Subjects in Japan and was approved by the institutional review board of our institution (approval number: 24-33).

### Clinicopathological data

The clinicopathological variables analyzed included age, gender, American Society of Anesthesiologists physical status (ASA-PS), tumor location, histological type, precedent stoma creation, prior bridge-to-surgery intervention, surgical emergency, surgical approach, adjuvant chemotherapy, pathological tumor depth (pT), nodal status (pN), lymphovascular invasion, and preoperative tumor markers (CEA and CA19-9).

ASA-PS was prospectively documented preoperatively by an anesthesiologist in all cases. Tumor laterality was defined anatomically; lesions located proximal to the splenic flexure were classified as right-sided tumors. A bridge-to-surgery procedure was defined as decompression by insertion of a self-expandable metallic stent or an ileus tube in patients presenting with obstructive CRC. A minimally invasive approach was defined as a laparoscopic and/or robotic procedure. Tumor staging was determined in accordance with the Union for International Cancer Control TNM classification system ^(9)^.

Serum CEA and CA19-9 were evaluated using standard institutional assays. The cutoff values were set at 5.0 ng/mL for CEA and 37 U/mL for CA19-9, which are the most commonly used thresholds. Patients were categorized as tumor marker–positive if either marker exceeded the predefined cutoff and as tumor marker–negative only when both values were below the thresholds.

### Calculative definitions

The NUn score was calculated using the following formula:

11.3894 + (0.005 × CRP [mg/L]) + (0.186 × WBC [10^9^/L]) − (0.174 × serum albumin [g/L]) ^(6)^

As a conventional inflammation-based marker derived from CRP and albumin, the Glasgow prognostic score (GPS) was also evaluated ^(10)^. Patients with both elevated CRP (>10 mg/L) and hypoalbuminemia (<35 g/L) were assigned a GPS of 2. Those with only one abnormal parameter were assigned a score of 1, whereas patients with neither abnormality were assigned a score of 0. All preoperative laboratory measurements were, in principle, obtained within 1 week prior to surgery.

### Postoperative follow-up

Patients were followed up at our outpatient clinics. Hematological tests and physical examinations were performed at least every 3 months for 5 years after surgery. Postoperative surveillance consisted of routine physical examinations, colonoscopy, computed tomography, abdominal ultrasonography, and standard blood testing at intervals in accordance with the guidelines of the Japanese Society for Cancer of the Colon and Rectum ^(11)^.

For patients who did not attend scheduled follow-up visits, survival and recurrence status were confirmed through structured telephone interviews. Follow-up for the entire cohort was completed in January 2026.

### Statistical analysis

Continuous variables were compared using the Wilcoxon rank-sum test. Overall survival (OS) was defined as the interval from the date of surgery to death from any cause, while relapse-free survival (RFS) was defined as the interval from surgery to either disease recurrence or death from any cause.

The optimal cutoff values for the NUn score were determined using X-tile software version 3.6.1 (Yale University School of Medicine, New Haven, CT, USA), which identifies the thresholds yielding the maximal separation of survival outcomes ^(12)^. Survival curves were constructed using the Kaplan-Meier method and compared with the log-rank test. The prognostic significance of each variable was initially assessed using univariate Cox proportional hazards regression analysis. Variables that were statistically significant in the univariate analysis, together with clinically relevant factors prespecified a priori, were subsequently entered into multivariate Cox proportional hazards models. Model discrimination and goodness-of-fit were evaluated using the Akaike information criterion (AIC) within the Cox proportional hazards framework.

All statistical tests were two-sided, and a p value <0.05 was considered statistically significant. Statistical analyses were performed using JMP Student Edition version 18.2.1 (SAS Institute Inc., Cary, NC, USA), X-tile, and R version 4.5.1 (R Foundation for Statistical Computing, Vienna, Austria).

## Results

### Associations between preoperative NUn score and clinicopathological features

The associations between the preoperative NUn score and clinicopathological variables are summarized in Table 1. The preoperative NUn score was significantly associated with several physiological, surgical, and pathological factors, including age at surgery (dichotomized at 70 years), ASA-PS, tumor location, histopathological type, prior bridge-to-surgery intervention, emergency surgery, surgical approach, pT, pN, lymphovascular permeation, and preoperative tumor marker positivity. In addition, increasing preoperative GPS was significantly correlated with higher NUn scores.

**Table 1.** Relationships between the Preoperative NUn Score and Clinicopathological Factors.

Variables		n	NUn score, median (IQR)	p-Value
Total		608	5.98 (5.37-6.85)	NA
Age at surgery (years)				0.001^*^
	≤69	203	5.71 (5.03-6.56)	
	≥70	405	6.06 (5.53-6.89)	
Gender				0.56
	Man	316	5.94 (5.36-6.75)	
	Woman	292	6.02 (5.39-6.86)	
ASA-PS				<0.001^*^
	1-2	509	5.87 (5.29-6.62)	
	3	99	6.52 (5.76-7.44)	
Main tumor location				<0.001^*^
	Right-sided colon	233	6.14 (5.58-7.09)	
	Left-sided colon	204	6.03 (5.38-6.90)	
	Rectum	171	5.67 (5.12-6.33)	
Main histology				<0.001^*^
	Differentiated	577	5.93 (5.36-6.71)	
	Undifferentiated	31	7.20 (5.97-8.84)	
Prior stoma creation				0.15
	Absent	586	5.97 (5.37-6.77)	
	Present	22	6.62 (5.40-7.47)	
Prior bridge-to-surgery intervention				<0.001^*^
	Absent	592	5.95 (5.36-6.74)	
	Present	16	6.96 (6.74-8.53)	
Emergency surgery				<0.001^*^
	No	591	5.95 (5.36-6.73)	
	Yes	17	8.63 (7.40-11.13)	
Minimally invasive approach				<0.001^*^
	No	409	6.28 (5.64-7.25)	
	Yes	199	5.54 (5.01-5.99)	
Adjuvant chemotherapy				0.18
	Absent	410	5.89 (5.35-6.75)	
	Present	198	6.09 (5.42-6.99)	
pT category				<0.001^*^
	T1-2	146	5.49 (5.00-5.94)	
	T3-4	462	6.19 (5.55-7.11)	
pN category				0.015^*^
	N0	381	5.80 (5.28-6.70)	
	N1-2	227	6.14 (5.53-7.00)	
Lymphovascular involvement				<0.001^*^
	Negative	243	5.73 (5.24-6.37)	
	Positive	365	6.14 (5.47-7.02)	
Preoperative tumor markers				<0.001^*^
	Negative	358	5.75 (5.25-6.49)	
	Positive	250	6.19 (5.59-7.26)	
Preoperative GPS				<0.001^*^
	0	397	5.60 (5.06-6.02)	
	1	131	6.92 (6.31-7.36)	
	2	80	8.35 (7.30-9.72)	

ASA-PS: American Society of Anesthesiologists physical status; GPS: Glasgow prognostic score; IQR: interquartile range; NA: not applicable; NUn: Noble and Underwood; pN: nodal status; pT: tumor depth. ^*^p < 0.05.

### Survival curve analysis

The median follow-up period among survivors was 65 months. During the study period, tumor recurrence occurred in 130 of the 608 patients (21.4%), of whom 73 subsequently died due to disease recurrence.

The optimal cutoff values for the preoperative NUn score were determined using X-tile software based on 5-year OS data. The optimal trichotomous thresholds were 5.60 and 7.12 (Figure 1). Patients were subsequently stratified into three groups according to these thresholds (low: ≤5.60; intermediate: >5.60 to ≤7.12; and high: >7.12), and Kaplan-Meier survival analyses were performed for OS (Figure 2) and RFS (Figure 3).

**Figure 1.**
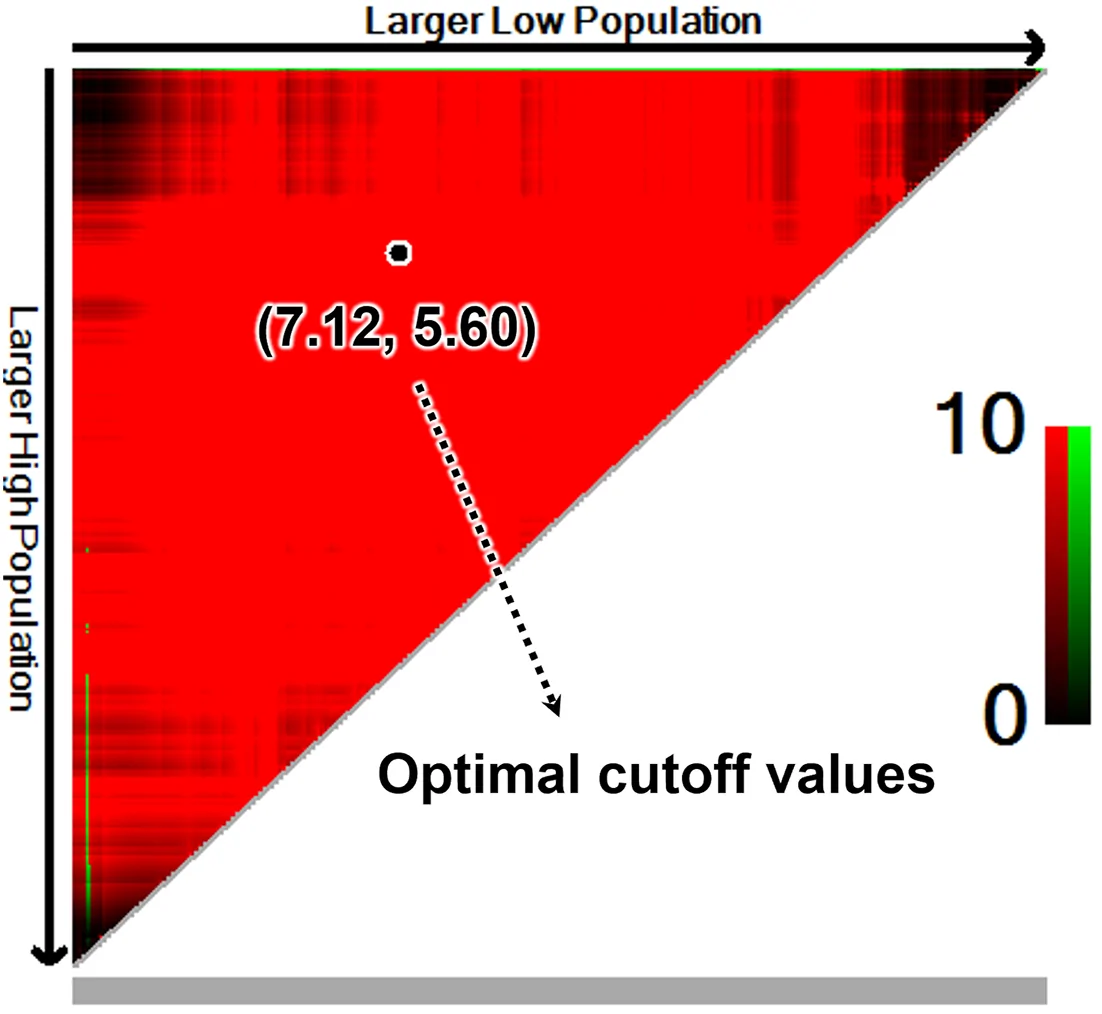
Data-driven cutoff determination of the preoperative NUn score using X-tile software. NUn: Noble and Underwood.

**Figure 2.**
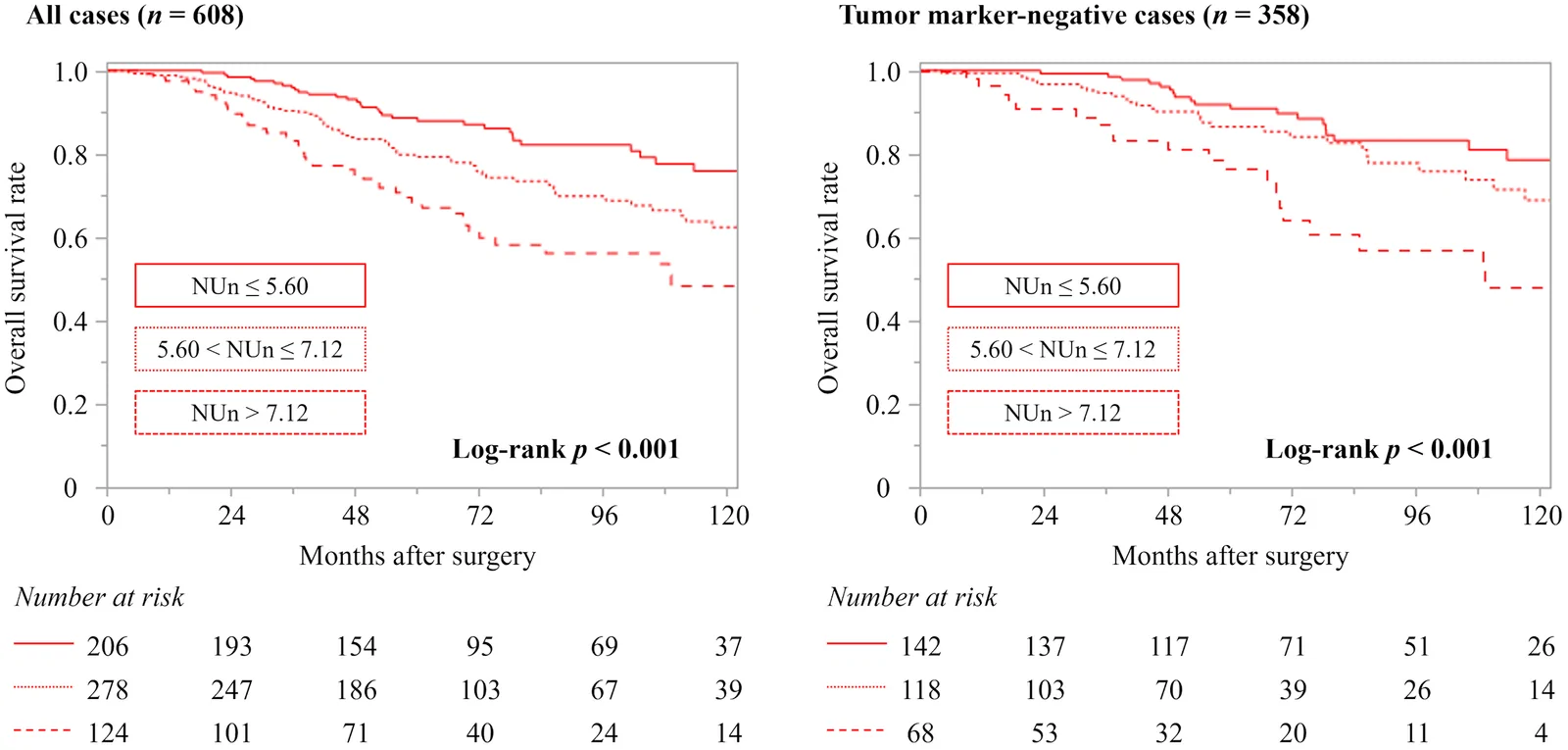
Overall survival curves according to the preoperative NUn score. NUn: Noble and Underwood.

**Figure 3.**
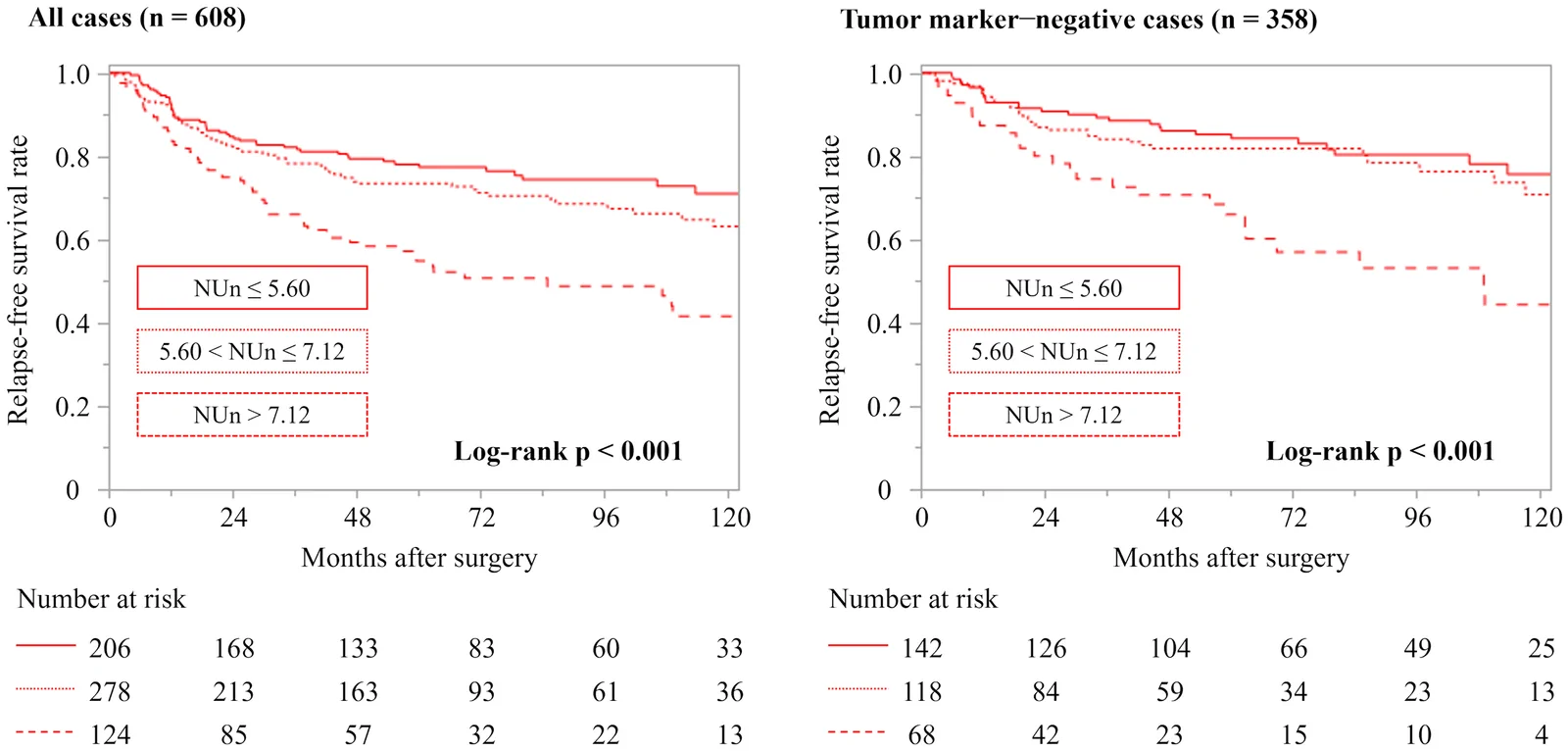
Relapse-free survival curves according to the preoperative NUn score. NUn: Noble and Underwood.

Both OS and RFS were explicitly stratified according to the NUn score. Patients with higher NUn scores exhibited significantly poorer survival outcomes. Notably, even among tumor marker–negative cases, in which conventional tumor markers are generally less informative for prognostic assessment, the NUn score successfully discriminated survival outcomes.

### Cox regression analyses

Table 2 presents the results of univariate Cox regression analyses for OS and RFS. The preoperative NUn score was significantly associated with both OS and RFS, whether analyzed as a trichotomous categorical variable or as a continuous variable. The preoperative GPS was also significantly associated with OS and RFS in univariate analyses. In both OS and RFS analyses, the trichotomous NUn model showed superior model fit compared with the GPS, as reflected by lower AIC values (OS: 1705.1 vs. 1719.9; RFS: 2284.4 vs. 2298.4). The concordance-index for OS was 0.62 for the NUn score and 0.56 for the GPS, while that for RFS was 0.58 and 0.54, respectively.

**Table 2.** Univariate Cox Regression Analyses for Overall and Relapse-Free Survival.

Variables		Overall survival		Relapse-free survival	
		HR (95% CI)	p-Value	HR (95% CI)	p-Value
Age at surgery (years)			0.002^*^		0.07
	≤69	1		1	
	≥70	1.81 (1.25-2.63)		1.33 (0.98-1.83)	
Gender			0.003^*^		0.002^*^
	Man	1		1	
	Woman	0.60 (0.43-0.84)		0.63 (0.47-0.85)	
ASA-PS			<0.001^*^		0.015^*^
	1-2	1		1	
	3	1.94 (1.34-2.83)		1.53 (1.09-2.15)	
Main tumor location			0.44		0.083
	Right-sided colon	1		1	
	Left-sided colon	1.18 (0.80-1.75)		1.36 (0.97-1.92)	
	Rectum	1.29 (0.87-1.91)		1.45 (1.02-2.06)	
Main histology			0.38		0.53
	Differentiated	1		1	
	Undifferentiated	0.64 (0.24-1.74)		0.78 (0.37-1.67)	
Prior stoma creation			0.12		0.016^*^
	Absent	1		1	
	Present	1.76 (0.86-3.59)		2.06 (1.15-3.70)	
Prior bridge-to-surgery intervention			0.10		0.015^*^
	Absent	1		1	
	Present	1.98 (0.87-4.49)		2.30 (1.18-4.50)	
Emergency surgery			0.038^*^		0.053
	No	1		1	
	Yes	2.38 (1.05-5.41)		2.02 (0.99-4.10)	
Minimally invasive approach			0.003^*^		<0.001^*^
	No	1		1	
	Yes	0.55 (0.37-0.81)		0.53 (0.38-0.75)	
Adjuvant chemotherapy			0.72		0.22
	Absent	1		1	
	Present	1.06 (0.76-1.49)		1.20 (0.90-1.61)	
pT category			0.002^*^		<0.001^*^
	T1-2	1		1	
	T3-4	1.99 (1.29-3.06)		1.94 (1.33-2.84)	
pN category			<0.001^*^		<0.001^*^
	N0	1		1	
	N1-2	2.14 (1.55-2.96)		2.37 (1.78-3.15)	
Lymphovascular involvement			0.002^*^		<0.001^*^
	Negative	1		1	
	Positive	1.76 (1.23-2.51)		1.88 (1.37-2.58)	
Preoperative tumor markers			<0.001^*^		<0.001^*^
	Negative	1		1	
	Positive	2.08 (1.50-2.87)		2.31 (1.74-3.08)	
Preoperative GPS			0.005^*^		0.020^*^
	0	1		1	
	1	1.70 (1.16-2.49)		1.49 (1.07-2.08)	
	2	1.74 (1.11-2.71)		1.53 (1.03-2.28)	
Preoperative NUn score (trichotomous)			<0.001^*^		<0.001^*^
	Low (≤5.60)	1		1	
	Intermediate (>5.60 to ≤7.12)	1.84 (1.21-2.79)		1.31 (0.92-1.86)	
	High (>7.12)	3.08 (1.96-4.84)		2.41 (1.66-3.51)	
Preoperative NUn score (continuous)			<0.001^*^		<0.001^*^
	+1-unit	1.19 (1.08-1.29)		1.18 (1.09-1.27)	

ASA-PS: American Society of Anesthesiologists physical status; CI: confidence interval; GPS: Glasgow prognostic score; HR hazard ratio; NUn: Noble and Underwood; pN: nodal status; pT: tumor depth. ^*^p < 0.05.

In multivariate Cox regression analysis for OS (Table 3), the preoperative NUn score remained an independent prognostic factor after adjustment for clinically relevant variables, including age, gender, ASA-PS, tumor sidedness, prior stoma creation, prior bridge-to-surgery intervention, emergency surgery, minimally invasive approach, adjuvant chemotherapy, pT, pN, lymphovascular involvement, and preoperative tumor marker positivity (high NUn: hazard ratio [HR] 2.06, 95% confidence interval [CI] 1.24-3.41, p = 0.016), whereas the preoperative GPS did not retain significance. This association persisted when the NUn score was modeled as a continuous variable (HR 1.15, 95% CI 1.02-1.29 per 1-unit increase, p = 0.022).

**Table 3.** Multivariate Cox Regression Analyses for Overall Survival.

Variables		Overall survival		
		HR (95% CI)^*^	AIC	p-Value
Preoperative GPS			1679.1	0.34
	0	1		
	1	1.19 (0.79-1.78)		
	2	1.43 (0.87-2.33)		
Preoperative NUn score (trichotomous)			1673.0	0.016^†^
	Low (≤5.60)	1		
	Intermediate (>5.60 to ≤7.12)	1.37 (0.88-2.12)		
	High (>7.12)	2.06 (1.24-3.41)		
Preoperative NUn score (continuous)			1674.1	0.022^†^
	+1-unit	1.15 (1.02-1.29)		

AIC: Akaike information criterion; ASA-PS: American Society of Anesthesiologists physical status; CI: confidence interval; GPS: Glasgow prognostic score; HR: hazard ratio; NUn: Noble and Underwood. *Adjusted with age (≥70 years), gender, ASA-PS (3), tumor location, prior stoma creation, prior bridge-to-surgery intervention, emergency surgery, minimally invasive approach, adjuvant chemotherapy, tumor depth (T3-4), nodal status, lymphovascular involvement, and preoperative tumor marker positivity. ^†^p < 0.05.

Similarly, the preoperative NUn score remained an independent predictor of RFS after adjustment for the aforementioned clinically relevant factors (high NUn: HR 1.66, 95% CI 1.09-2.53, p = 0.011) (Table 4). This association remained significant even when the NUn score was analyzed as a continuous variable (HR 1.14, 95% CI 1.02-1.26 per 1-unit increase, p = 0.015). According to the AIC, multivariate models with the NUn score showed better fit than those with the GPS. The full multivariable model including all covariates is presented in Supplementary Table S1.

**Table 4.** Multivariate Cox Regression Analyses for Relapse-Free Survival.

Variables		Relapse-free survival		
		HR (95% CI)^*^	AIC	p-Value
Preoperative GPS			2238.8	0.59
	0	1		
	1	1.00 (0.70-1.43)		
	2	1.24 (0.80-1.92)		
Preoperative NUn score (trichotomous)			2231.2	0.011^†^
	Low (≤5.60)	1		
	Intermediate (>5.60 to ≤7.12)	1.00 (0.69-1.45)		
	High (>7.12)	1.66 (1.09-2.53)		
Preoperative NUn score (continuous)			2232.1	0.015^†^
	+1-unit	1.14 (1.02-1.26)		

AIC: Akaike information criterion; ASA-PS: American Society of Anesthesiologists physical status; CI: confidence interval; GPS: Glasgow prognostic score; HR: hazard ratio; NUn: Noble and Underwood. *Adjusted with age (≥70 years), gender, ASA-PS (3), tumor location, prior stoma creation, prior bridge-to-surgery intervention, emergency surgery, minimally invasive approach, adjuvant chemotherapy, tumor depth (T3-4), nodal status, lymphovascular involvement, and preoperative tumor marker positivity. ^†^p < 0.05.

## Discussion

CRC is widely recognized as exhibiting marked prognostic heterogeneity, with substantial variation in clinical outcomes even among patients with similar pathological stage or conventional risk factors. This complexity limits the predictive capacity of traditional staging systems and tumor markers, underscoring the need for more refined and biologically informative prognostic models.

In this analysis of 608 patients with resectable CRC, we demonstrated that the preoperative NUn score, a simple laboratory-based index, provides robust prognostic discrimination. To the best of our knowledge, this is the first report to establish the prognostic relevance of the NUn score in CRC. Given the extensive prior investigation into inflammation-based prognostic markers in this field, our findings introduce a novel and clinically practical perspective. Notably, the NUn score retained its discriminative ability even among tumor marker–negative cases, a population in which conventional tumor markers offer limited prognostic guidance. This finding is clinically meaningful, as it suggests that the NUn score may assist in therapeutic stratification even in patients for whom risk assessment is otherwise challenging.

The NUn score was originally described in 2011 by Noble and Underwood as a logistic regression–based model incorporating systemic inflammatory blood markers to predict anastomotic leakage and major postoperative complications after esophagectomy ^(6)^. The biological plausibility of the NUn score is supported by the well-established relationship between systemic inflammation and tumor progression. Each component of the NUn score—WBC count, CRP, and serum albumin—is well recognized as an inflammation-related parameter. Chronic inflammation drives tumor progression by promoting proliferation, vascular endothelial growth factor–mediated angiogenesis, immunosuppression, and metastatic dissemination ^(13)^. Accordingly, a high NUn score may represent a protumorigenic inflammatory milieu. Elevated CRP and hypoalbuminemia can be interpreted as manifestations of cancer-related systemic inflammatory response and may indirectly reflect tumor burden ^(14)^. Hypoalbuminemia is also associated with impaired immune competence and overall physiological vulnerability ^(15)^. The combination of WBC, CRP, and serum albumin may enable multidimensional assessment of inflammatory magnitude, persistence, and host response, thereby explaining its favorable prognostic performance. Indeed, in this study, the NUn score outperformed the conventional GPS, which is based solely on CRP and albumin. Although the NUn score was significantly associated with both OS and RFS, its particularly strong discrimination for RFS suggests a close link with oncological disease activity. This score was originally developed to predict postoperative complications following esophageal surgery ^(6)^, and whether the coefficients included in the formula are optimally calibrated for CRC remains uncertain. Nevertheless, its reported utility in other malignancies, including gastric and lung cancers ^(7), (8)^, suggests a degree of potential generalizability.

From a clinical perspective, the NUn score should be positioned not as a stand-alone determinant of treatment but as a practical risk-modifying tool to optimize therapeutic intensity and surveillance strategies. It may aid in tailoring adjuvant therapy in borderline stage II/III cases, strengthening perioperative nutritional and exercise interventions (prehabilitation), and individualizing follow-up intensity according to recurrence risk ^(16), (17)^. Although the biological basis of its prognostic impact remains to be fully elucidated, whether reflecting tumor-driven inflammation, host vulnerability, comorbidity burden, or their interplay, prospective validation and tumor-specific predictive evidence are still required before routine incorporation into standard CRC management can be justified.

Several limitations should be acknowledged. First, this was a single-institution retrospective study, and our findings may represent chance observations within this cohort. Second, concomitant inflammatory conditions may have influenced the assessment of the NUn score; preoperative inflammatory comorbidities were not completely excluded, potentially affecting the optimal cutoff values. In particular, among patients undergoing emergency surgery or those receiving bridge-to-surgery treatment, host inflammatory status may substantially influence the NUn score. While multivariable analyses were performed to mitigate this effect, caution is still warranted in interpreting the results. Third, given that patient data were collected over more than a decade (2009-2022), temporal bias related to evolving surgical techniques, perioperative management, and systemic therapies cannot be excluded. Fourth, the cutoff values were derived from the same dataset, potentially leading to overfitting and limiting generalizability. Rigorous external validation in independent cohorts is therefore essential to confirm the stability and reproducibility of these findings. Finally, while the NUn score was compared with the conventional prognostic indicator GPS in this study, comparisons with other established scoring systems, such as the prognostic nutritional index, were not performed. Consequently, the relative positioning of the NUn score among existing prognostic factors remains to be clarified.

In summary, the preoperative NUn score was an independent predictor of both overall OS and RFS in patients with resectable colorectal cancer. This prognostic association remained evident even among tumor marker–negative cases. The stable and reproducible prognostic performance of the NUn score suggests its potential utility as a risk-modifying tool to support personalized therapeutic decision-making and surveillance strategies.

## Article Information

### Author Contributions

Masayuki Urabe contributed to the conception and design of the study. All authors acquired data. Masayuki Urabe performed data interpretation and drafted the manuscript. Yoshitaka Kiya, Goki Morizono, Masaya Hiyoshi, Toshiyuki Watanabe, and Yojiro Hashiguchi critically revised the manuscript. All authors read and approved the final version prior to submission.

### Conflicts of Interest

None

### Ethics Approval

This study was approved by the institutional ethics committee of the Japanese Red Cross Omori Hospital (approval number: 24-33).

### Informed Consent

Written informed consent was waived because of the retrospective design.

## Supplement

Supplementary Material
