# Supplementary material for: Preoperative Noble and Underwood Score in Colorectal Cancer Surgery: A Stable Prognostic Marker Even among Tumor Marker–Negative Cases
Source: JMA J. 2026 Jul 3;9(5):1198–206. doi: 10.31662/jmaj.2026-0107 (PMC13639637; doi:10.31662/jmaj.2026-0107)
Supplement: Supplementary file 1 [file 2433-3298-9-5-1198-s001.pdf]

Supplementary Table S1. Full Multivariable Cox Regression Models for Overall and Relapse-Free Survivals.

Model for preoperative GPS				
			[AIC] OS 1679.1; RFS 2238.8	
**Variables**	**Overall survival**		**Relapse-free survival**	
	**HR (95% CI)**	**p-Value**	**HR (95% CI)**	**p-Value**
Age at surgery (years)		0.005*		0.081
≤69	1		1	
≥70	1.76 (1.18-2.63)		1.35 (0.96-1.89)	
Gender		<0.001*		0.002*
Man	1		1	
Woman	0.55 (0.38-0.78)		0.62 (0.46-0.84)	
ASA-PS		0.079		0.45
1-2	1		1	
3	1.44 (0.96-2.17)		1.15 (0.80-1.67)	
Main tumor location		0.10		0.021*
Right-sided colon	1		1	
Left-sided colon	1.09 (0.73-1.65)		1.31 (0.92-1.87)	
Rectum	1.55 (1.02-2.35)		1.69 (1.17-2.46)	
Prior stoma creation		0.49		0.17
Absent	1		1	
Present	1.30 (0.62-2.76)		1.55 (0.84-2.86)	
Prior bridge-to-surgery intervention		0.50		0.10
Absent	1		1	
Present	1.34 (0.58-3.13)		1.82 (0.90-3.67)	
Emergency surgery		0.041*		0.11
No	1		1	
Yes	2.50 (1.04-6.01)		1.87 (0.87-4.01)	
Minimally invasive approach		0.44		0.27
No	1		1	
Yes	0.84 (0.54-1.31)		0.80 (0.54-1.18)	
Adjuvant chemotherapy		0.030*		0.038*
Absent	1		1	
Present	0.65 (0.44-0.96)		0.69 (0.49-0.98)	

pT category		0.37		0.58
T1-2	1		1	
T3-4	1.25 (0.76-2.07)		1.13 (0.73-1.76)	
pN category		<0.001*		<0.001*
N0	1		1	
N1-2	2.00 (1.37-2.94)		2.15 (1.54-3.01)	
Lymphovascular involvement		0.50		0.30
Negative	1		1	
Positive	1.16 (0.76-1.76)		1.22 (0.84-1.76)	
Preoperative tumor markers		0.010*		<0.001*
Negative	1		1	
Positive	1.58 (1.12-2.23)		1.77 (1.30-2.40)	
Preoperative GPS		0.34		0.59
0	1		1	
1	1.19 (0.79-1.78)		1.00 (0.70-1.43)	
2	1.43 (0.87-2.33)		1.24 (0.80-1.92)	

Model for preoperative NUn score (trichotomous)

[AIC] OS 1673.0; RFS 2231.2

Variables	Overall survival		Relapse-free survival	
	**HR (95% CI)**	**p-Value**	**HR (95% CI)**	**p-Value**
Age at surgery (years)		0.012*		0.10
≤69	1		1	
≥70	1.68 (1.12-2.51)		1.33 (0.94-1.86)	
Gender		<0.001*		0.003*
Man	1		1	
Woman	0.55 (0.39-0.79)		0.63 (0.46-0.85)	
ASA-PS		0.13		0.69
1-2	1		1	
3	1.38 (0.91-2.07)		1.08 (0.74-1.57)	
Main tumor location		0.085		0.015*
Right-sided colon	1		1	
Left-sided colon	1.14 (0.76-1.71)		1.32 (0.93-1.88)	
Rectum	1.58 (1.05-2.40)		1.72 (1.19-2.49)	
Prior stoma creation		0.57		0.26

Absent	1		1	
Present	1.24 (0.59-2.63)		1.42 (0.77-2.63)	
Prior bridge-to-surgery intervention		0.41		0.062
Absent	1		1	
Present	1.43 (0.62-3.32)		1.93 (0.97-3.87)	
Emergency surgery		0.072		0.21
No	1		1	
Yes	2.24 (0.93-5.39)		1.63 (0.76-3.48)	
Minimally invasive approach		0.62		0.40
No	1		1	
Yes	0.89 (0.57-1.40)		0.85 (0.57-1.25)	
Adjuvant chemotherapy		0.033*		0.047*
Absent	1		1	
Present	0.65 (0.44-0.97)		0.70 (0.50-0.99)	
pT category		0.47		0.66
T1-2	1		1	
T3-4	1.20 (0.73-1.99)		1.11 (0.71-1.72)	
pN category		<0.001*		<0.001*
N0	1		1	
N1-2	1.93 (1.32-2.83)		2.13 (1.52-2.97)	
Lymphovascular involvement		0.57		0.35
Negative	1		1	
Positive	1.13 (0.74-1.72)		1.19 (0.82-1.72)	
Preoperative tumor markers		0.011*		<0.001*
Negative	1		1	
Positive	1.57 (1.11-2.22)		1.75 (1.28-2.38)	
Preoperative NUn score		0.016*		0.011*
Low (≤5.60)	1		1	
Intermediate (>5.60 to ≤7.12)	1.37 (0.88-2.12)		1.00 (0.69-1.45)	
High (>7.12)	2.06 (1.24-3.41)		1.66 (1.09-2.53)	

**Model for preoperative NUn score (continuous)** [AIC] OS 1674.1; RFS 2232.1				
**Variables**	**Overall survival**		**Relapse-free survival**	
	**HR (95% CI)**	**p-Value**	**HR (95% CI)**	**p-Value**

Age at surgery (years)		0.004*		0.074
≤69	1		1	
≥70	1.79 (1.20-2.66)		1.36 (0.97-1.90)	
Gender		<0.001*		0.003*
Man	1		1	
Woman	0.55 (0.38-0.78)		0.63 (0.47-0.86)	
ASA-PS		0.12		0.61
1-2	1		1	
3	1.39 (0.92-2.09)		1.10 (0.76-1.60)	
Main tumor location		0.079		0.012*
Right-sided colon	1		1	
Left-sided colon	1.10 (0.73-1.65)		1.32 (0.93-1.88)	
Rectum	1.59 (1.05-2.41)		1.76 (1.21-2.56)	
Prior stoma creation		0.51		0.20
Absent	1		1	
Present	1.29 (0.61-2.72)		1.49 (0.81-2.74)	
Prior bridge-to-surgery intervention		0.64		0.13
Absent	1		1	
Present	1.23 (0.52-2.90)		1.72 (0.86-3.45)	
Emergency surgery		0.096		0.31
No	1		1	
Yes	2.14 (0.87-5.24)		1.51 (0.68-3.35)	
Minimally invasive approach		0.54		0.43
No	1		1	
Yes	0.87 (0.56-1.36)		0.86 (0.58-1.26)	
Adjuvant chemotherapy		0.022*		0.034*
Absent	1		1	
Present	0.63 (0.43-0.93)		0.69 (0.49-0.97)	
pT category		0.44		0.73
T1-2	1		1	
T3-4	1.22 (0.74-2.01)		1.08 (0.69-1.68)	
pN category		<0.001*		<0.001*
N0	1		1	
N1-2	2.06 (1.40-3.02)		2.18 (1.56-3.04)	
Lymphovascular involvement		0.56		0.35
Negative	1		1	

Positive	1.14 (0.74-1.73)		1.19 (0.82-1.72)	
Preoperative tumor markers		0.009*		0.003*
Negative	1		1	
Positive	1.59 (1.12-2.25)		1.77 (1.30-2.41)	
Preoperative NUn score		0.022*		0.015*
+1-unit	1.15 (1.02-1.29)		1.14 (1.02-1.26)	

AIC: Akaike information criterion; ASA-PS: American Society of Anesthesiologists physical status; CI: confidence interval; GPS: Glasgow prognostic score; HR: hazard ratio; NUn: Noble and Underwood; OS: overall survival; pN: nodal status; pT: tumor depth; RFS: relapse-free survival.

*$p < 0.05$.
